# American Medical Association

**Published:** 1881-05-20

**Authors:** 


### *AMERICAN MEDICAL ASSOCIATION.*

The Association assembled in Richmond, Va., on the 3d inst., at Mozart Hall. The address of welcome was made by his Excellency, Governor Holliday, of the State of Virginia. His address was brief, but cordial and complimentary, containing the following utterance: "Of all the conventions that ever here met, none in the hight and nobility of their aims surpassed that into the faces of whose members I am now looking."

Dr. John T. Hodgen, of Missouri, President, made a lengthy and able address.

The officers elected for the ensuing year are as follows:

*President*—Dr. T. J. Woodward, U. S. A.

*Vice-Presidents*—Dr. P. O. Harper, Arkansas; Dr. L. Connor, Michigan; D.. Eugene Gresson, North Carolina; Dr. Hunter McGuire, Virginia.

*Secretary*—Dr. Wm. B. Atkinson, Pennsylvania.

*Treasurer*—Dr. R. J. Dunglison, Pennsylvania.

*Librarian*—Dr. Wm. Lee, Washington.

St. Paul, Minnesota, was selected as the place for the next annual meeting.

The various *Sections* assembled in their separate capacities, and the usual number of able and interesting papers were read.

The report on Journalizing Transactions recommended that a committee of five be appointed, whose duty it shall be to digest and report in detail, as early as possible, a plan for the publication of a Weekly Journal by the Association; the nomination of an Editor, his salary, and the time and place of publication of such Journal.

The following amendment to the Code of Ethics, after considerable discussion, was adopted:

"It is not in accord with the interest of the public or the honor of the profession, that any physician or medical teacher should examine or sign diplomas or certificates of proficiency for, or otherwise be specially concerned with, the graduation of persons whom they have good reason to believe intend to support and practice any exclusive and irregular system of medicine."

The following are the officers of Sections for ensuing year, to-wit:

*Section on Practice of Medicine:* Chairman, Dr. J. A. Octerberry, Kentucky; Secretary, Dr. Robert J. Roberts, Tennessee.

*Section on Surgery and Anatomy:* Chairman, Dr. J. C. Hughes, Iowa; Secretary, Dr. William A. Byrd, Illinois.

*Section on Obstetrics:* Chairman, Dr. H. O. Marcy, Massachusetts; Secretary, Dr. C. V. Mottram, Kansas.

*Section on Medical Jurisprudence and State Medicine:* Chairman, Dr. A. L. Gihon, Washington, D. C.; Secretary, Dr. J. H. Sears, Texas.

*Ophthalmology, Otology, and Laryngology:* Chairman, Dr. D. B. St. John Roosa, New York; Secretary, J. Sales Cohen, Philadelphia, Pa.

*Diseases of Children:* Chairman, Dr. S. C. Busy, Washington, D. C.; Secretary, Dr. William Lee, Baltimore, Md.

*Dentistry:* Chairman, Dr. D. H. Goodville, New York; Secretary, Dr. P. W. Brophy, Illinois.

*Judicial Council:* Drs. S. N. Benham, Pennsylvania; John Toner, Washington, D. C.; D. A. Linthineum, Arkansas; William Brodie, Michigan; H. S. Holton, Vermont; A. B. Sloan, Missouri; R. Beverly Cole, California.

In reference to trade-marks, copyrights, etc., the following was offered, and referred to the judicial council to be reported next year:

*Resolved,* That the spirit of the Code of Ethics forbids a physician from prescribing a remedy controlled by a patent, copyright or trademark. This, however, shall except a patent upon a process of manufacture or upon the machinery of the manufacture, provided the patent be not used to prevent legitimate competition, and shall also except the use of a trade-mark used to designate a brand of manufacture, provided that the article so marked be accompanied by working formula duly sworn to, and also by a technical name under which any one can compete in the manufacture of the same.

The attendance was perhaps an average one in all respects. The largest delegations were from Pennsylvania, Virginia, and New York.

Our own State, Georgia, was represented by the following gentlemen: Thomas S. Powell, Alban S. Payne, W. P. Nicolson, of Atlanta—H. F. Campbeli, of Augusta, and Robert Battey, of Rome.

The next meeting will be held at St. Paul, Minnesota.

